# Adoptive immunotherapy with MUC1-mRNA transfected dendritic cells and cytotoxic lymphocytes plus gemcitabine for unresectable pancreatic cancer

**DOI:** 10.1186/1479-5876-12-175

**Published:** 2014-06-19

**Authors:** Yoshitaro Shindo, Shoichi Hazama, Yoshinari Maeda, Hiroto Matsui, Michihisa Iida, Nobuaki Suzuki, Kiyoshi Yoshimura, Tomio Ueno, Shigefumi Yoshino, Kohei Sakai, Yutaka Suehiro, Takahiro Yamasaki, Yuji Hinoda, Masaaki Oka

**Affiliations:** 1 Department of Digestive Surgery and Surgical Oncology (Department of Surgery II), Yamaguchi University Graduate School of Medicine, 1-1-1 Minami-kogushi, Ube, Yamaguchi 755-8505, Japan; 2 Department of Oncology and Laboratory Medicine, Yamaguchi University School of Medicine, 1-1- 1 Minami-Kogushi, Ube, Yamaguchi 755-8505, Japan

**Keywords:** Pancreatic cancer, MUC1, Dendritic cell, Cytotoxic lymphocyte, Gemcitabine, Immunotherapy

## Abstract

**Background:**

We previously reported the clinical efficacy of adoptive immunotherapy (AIT) with dendritic cells (DCs) pulsed with mucin 1 (MUC1) peptide and cytotoxic T lymphocytes (CTLs). We also reported that gemcitabine (GEM) enhances anti-tumor immunity by suppressing regulatory T cells. Therefore, in the present study, we performed combination therapy with AIT and GEM for patients with unresectable or recurrent pancreatic cancer.

**Patients and methods:**

Forty-two patients with unresectable or recurrent pancreatic cancer were treated. DCs were generated by culture with granulocyte macrophage colony-stimulating factor and interleukin-4 and then exposed to tumor necrosis factor-α. Mature DCs were transfected with MUC1-mRNA by electroporation (MUC1-DCs). MUC1-CTLs were induced by co-culture with YPK-1, a human pancreatic cancer cell line, and then with interleukin-2. Patients were treated with GEM, while MUC1-DCs were intradermally injected, and MUC1-CTLs were intravenously administered.

**Results:**

Median survival time (MST) was 13.9 months, and the 1-year survival rate was 51.1%. Of 42 patients, one patient had complete response (2.4%), three patients had partial response (7.1%) and 22 patients had stable disease (52.4%). The disease control ratio was 61.9%. The MST and 1-year survival rate of 35 patients who received more than 1 × 10^7^ MUC1-DCs per injection was 16.1 months and 60.3%, respectively. Liver metastasis occurred in only 5 patients among 35 patients without liver metastasis before treatment. There were no severe toxicities associated with AIT.

**Conclusion:**

AIT with MUC1-DCs and MUC1-CTLs plus GEM may be a feasible and effective treatment for pancreatic cancer.

## Background

Pancreatic adenocarcinoma is the fourth leading cause of cancer death worldwide and has an overall 5-year survival rate of only 6% [1]. No adequate therapy for pancreatic cancer has yet been found, and most patients die within a year of diagnosis. New treatment strategies are therefore necessary.

Immunotherapy has an advantage over radiation and chemotherapies because it can act specifically against the tumor without damaging normal tissue. Immunotherapeutic approaches to pancreatic cancer have included the use of monoclonal antibodies (mAbs) [2], cytokines [3], vaccines [4], and lymphokine-activated killer (LAK) cells [5]. Dendritic cells (DCs) play important roles as antigen-presenting cells in innate and adaptive immunity [6]. DC-based therapy has been used in clinical trials for various cancers including pancreatic cancer [7-9].

Mucin 1 (MUC1) is overexpressed in an incompletely glycosylated form in various human cancers [10]. We have previously reported that the expression of MUC1 was observed in all cancer cells from all 55 pancreatic ductal adenocarcinomas as well as 2 liver metastases by immunohistochemistry. In contrast the expression of MUC1 was not observed in specimens from normal pancreas, chronic pancreatitis, or ductal hyperplasia of the pancreas [11]. Cytotoxic T lymphocytes (CTLs) recognize MUC1 molecules in a human leukocyte antigen (HLA)-unrestricted manner, which means that these cells can be used for all cancer patients such as pancreatic, breast, or ovarian cancer expressing MUC1 antigen [12-16]. We have previously reported adoptive immunotherapy (AIT) with CTLs stimulated by a MUC1-expressing human pancreatic cancer cell line, YPK-1, (MUC1-CTLs) for unresectable pancreatic cancer [15]. We have also reported the efficacy of AIT with MUC1 peptide-pulsed DCs and MUC1-CTLs [16].

DCs are potent antigen-presenting cells for induction of primary T-cell dependent immune responses [6]. Numerous studies have demonstrated the feasibility of DC-based immunization to induce host responses against tumors [17]. DCs can be pulsed with peptide [16], full-length protein [18], or whole tumor cells [19], and transfected with DNA or RNA [20,21] or transduced with recombinant viruses [22]. Comparative studies suggest that mRNA transfection may be superior to other antigen-loading techniques in generating immunocompetent DCs [23].

Gemcitabine (GEM), which is a standard chemotherapeutic agent for pancreatic cancer [24], is not immunosuppressive and may enhance responses to specific vaccines or immunotherapy administered to activate or support immune responses directed toward driving effector immunity to cancer cells [24,25]. Treatment with GEM sensitizes human pancreatic carcinoma cell lines against CTL-mediated lysis [26]. DC-based vaccination combined with GEM increases survival in a murine pancreatic carcinoma model [27].

To create a more effective therapy for pancreatic cancer, we conducted combined AIT with MUC1-CTLs and MUC1-mRNA–transfected dendritic cells (MUC1-DCs) plus GEM.

## Patients and methods

### Patients and eligibility criteria

Between 2007 and 2012, 42 patients with unresectable or recurrent pancreatic cancer histologically confirmed as invasive ductal carcinoma by endoscopic ultrasound-guided fine-needle aspiration were treated at the Department of Digestive Surgery and Surgical Oncology (Department of Surgery II) of the Yamaguchi University Graduate School of Medicine. This therapy was not a clinical trial, but a medical treatment approved as advanced health care by the Japanese Ministry of Health, Labor and Welfare, and provided for all patients who could pay the cost for this therapy and who met the basic criteria as described below. We retrospectively summarized safety and efficacy of this therapy. The study protocol was also approved by the Institutional Review Board for Human Use at the Yamaguchi University School of Medicine. Written informed consent was obtained from all patients.

Eligibility criteria were as follows: age of ≥20 years; life expectancy ≥3 months; and adequate hepatic, renal, and bone marrow function (serum creatinine level, <2.0 mg/dl; bilirubin level, <3.0 g/dl; platelet count, ≥75,000/ml; total white blood cell count ≥3,000/ml and ≤15,000/ml). All patients had to have an Eastern Cooperative Oncology Group (ECOG) performance status (PS) of 0–2 at the time of initial consultation.

### Treatment protocol

**Figure 1:**
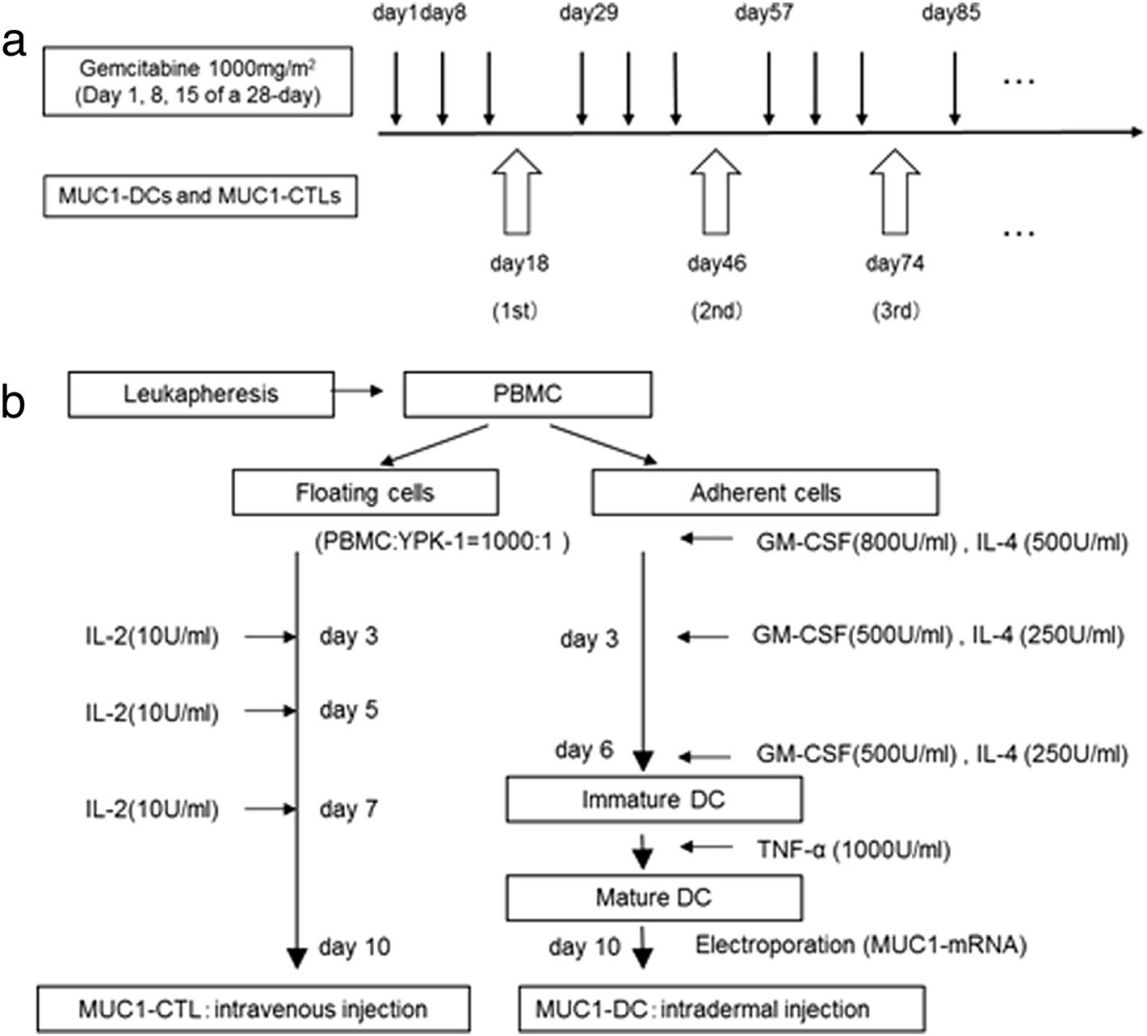
**Treatment regimen. a**; Patients were treated with GEM (1000 mg/m^2^) for 3 weeks (on days 1, 8, and 15) followed by 1 week of rest, while MUC1-DCs and MUC1-CTLs were administrated on day 18 every 4 weeks. **b**; MUC1-CTLs were induced by co-culture with YPK-1, a human pancreatic cancer cell line, and then with IL-2. DCs were generated by culture with GM-CSF and IL-4 and then exposed to TNF-α. Mature DCs were transfected with MUC1-mRNA by electroporation (MUC1-DCs).

Patients were treated with GEM (1000 mg/m^2^) for 3 weeks (on days 1, 8, and 15) followed by 1 week of rest, while MUC1-DCs suspended in 2 ml saline were injected intradermally in the inguinal region as maximum available cell products, and MUC1-CTLs suspended in 100 ml saline were given intravenously as maximum available cell products on day 18 every 4 weeks (Figure 1a). This AIT was repeated until progressive disease (PD) was recognized.

### Adverse events and clinical responses

Adverse events were evaluated according to the Common Terminology Criteria for Adverse Events v3.0 (CTCAE) [28]. Computed tomography (CT) scan or magnetic resonance imaging (MRI) examination was made before the treatment. Tumors were staged with the UICC classification system.

CT or MRI was made after the first 3 transfers and was repeated every 4 to 6 weeks after the treatment. Patients were assigned a response category according to the Response Evaluation Criteria in Solid Tumors (RECIST) Committee [29].

### Generation of MUC1-mRNA

MUC1-mRNA was transcribed in vitro. An XhoI fragment containing a full length of MUC1 cDNA was cloned into the XhoI site of the pcDNA3.1. Clones containing the MUC1 cDNA were isolated, and midi scale cDNA preparations were generated using Quantum Prep™ Plasmid Midiprep Kit (Bio Rad, Hercules, CA, USA). The plasmid vector was linearized with *Xho*I digest and purified with Wizard SV Gel and PCR Clean-Up System (Promega, Madison, Wisc., USA). In vitro transcription was then carried out using a mMessage mMachine® T7 Ultra Kit (Ambion, Austin, Tex., USA) according to the manufacturer’s protocol.

### Separation of adherent and non-adherent cells

Peripheral blood mononuclear cells (PBMCs) were harvested with the COBE Spectra Apheresis System (COBE BCT, Inc., Lakewood, CO, USA). PBMCs from 3000 ml of blood were enriched by density gradient centrifugation with Ficoll-Paque (Amersham Pharmacia Biotech, Uppsala, Sweden). The PBMCs were incubated for 45 min in a 5% CO_2_ atmosphere at 37°C in serum-free AIM-V medium (Gibco, Paisley, Scotland). Plastic-adherent cells were used for the generation of DCs, while non-adherent cells were used for the generation of CTLs.

### Generation of MUC1-CTLs

MUC1-CTLs were induced as previously described [15]. Briefly, non-adherent cells were cultured in AIM-V with the MUC1-expressing human pancreatic cancer cell line YPK-1 (HLA-A2402) inactivated with 0.2 mg/ml mitomycin C (Kyowa Hakko Kogyo Co., Ltd., Tokyo, Japan). The effector-to-stimulator cell ratio was 1,000:1. On days 3, 5, and 7, recombinant human interleukin 2 (IL-2) (Shionogi Pharmaceutical Co., Tokyo, Japan) was added to the cultures at a final concentration of 10 units/ml (U/ml). The plates were incubated in a 5% CO_2_ atmosphere at 37°C. On day 10, MUC1-CTLs were washed 3 times with saline, suspended in 100 ml saline and administered intravenously (Figure 1b).

### Cytotoxicity assay and antibody inhibition assay of cytotoxicity

Cytotoxicity assays of MUC1-CTLs induced from a healthy volunteer with the HLA-A 24/26 were performed as previously described (Additional file 1: Figure S1a) [15]. Briefly, target cells (1 × 10^6^/ml) were labeled for 60 min at 37˚C with 100 μCi/ml radioactive sodium chromate (^51^Cr) (Amersham Japan, Tokyo, Japan). The cells were then washed 4 times in RPMI-1640 medium (Sigma-Aldrich). Labeled cells were resuspended in culture medium (1 × 10^5^/ml). Effector cells consisting of induced MUC1-CTLs were suspended at 0.5, 1.0 or 2.0 × 10^6^/ml. Effector cell suspension (0.1 ml) was added to a microplate (Falcon Plastics, Cockeysville, MD) with 0.1 ml target cells, to yield an effector to target cell ratio of 5:1, 10:1 or 20:1. All experiments were performed in triplicate. Plates were incubated for 4 h at 37˚C in a CO_2_ incubator. The amount of ^51^Cr released into each well was determined with a γ counter (Auto Well Gamma System ARC-202, Aloka, Tokyo, Japan). The percentage of cytotoxicity was calculated as follows:

$$\%\;\mathrm{cytotoxicity}=\frac{\mathrm{experimental}\;\mathrm{release}\;‒\;\mathrm{spontaneous}\;\mathrm{release}}{\mathrm{maximum}\;\mathrm{release}\;‒\;\mathrm{spontaneous}\;\mathrm{release}}$$

To measure the spontaneous ^51^Cr release of target cells in the absence of effector cells, target cells were mixed with 0.1 ml culture medium. To obtain maximal a ^51^Cr release, target cells were treated with 0.1 ml 0.1 N hydrochloric acid [15].

Antibody inhibition assay of cytotoxicity of induced MUC1-CTLs induced from a healthy volunteer with the HLA-A 24/26 was described previously (Additional file 1: Figure S1b) [15]. Briefly, anti-CD3, -CD4, -CD8 and anti-class I mAbs (each diluted at 1:50) were used for blocking assays and were purchased from Dako Corp., Carpinteria, CA. The MUC1-expressing pancreatic cancer cell line YPK-1 was used as the target cell. Effector cells consisting of induced MUC1-CTLs were incubated with mAb at the indicated concentrations for 45 min at 37˚C, washed 3 times in RPMI-1640 medium and suspended at 2 × 10^6^/ml. Target cells were labeled with ^51^Cr as described above, washed 4 times and resuspended in culture medium (1 × 10^5^/ml). Effector cell suspension (0.1 ml) was added with 0.1 ml target cells to yield a 20:1 effector to target cell ratio for cytotoxicity assays as described above. For anti-MUC1 mAb blocking, target cells were preincubated for 1 h at 37˚C with anti-MUC1 mAb MY.1E12 (diluted at 1:200), kindly provided by Dr Tatsuo Irimura, Department of Cancer Biology and Molecular Immunology, Faculty of Pharmaceutical Sciences, University of Tokyo, Japan. Cytotoxicity assays were performed as described above [15].

### Generation of MUC1-DCs

Adherent cells were cultured in AIM-V medium containing 800 U/ml granulocyte macrophage colony-stimulating factor (GM-CSF) (Osteogenetics GmbH, Wurzburg, Germany) and 500 U/ml IL-4 (Osteogenetics GmbH). On days 3 and 6, GM-CSF and IL-4 were added to the cultures at a final concentration of 400 U/ml and 250 U/ml respectively. On day 6, immature DCs (imDCs) were cultured in AIM-V medium containing 1000 U/ml tumor necrosis factor-α (TNF-α) (R&D Systems, Minneapolis, MN, USA). On day 10, floating and loosely adherent cells were collected as mature DCs (mDCs). The mDCs were washed once, suspended in AIM-V medium, and adjusted to a final cell density of 2 × 10^6^ cells/ml. Subsequently, 400 μl of the cell suspension was mixed with 10 μg of MUC1-mRNA and electroporated in a 4-mm cuvette by using a BTX 830 square-wave electroporator (Harvard Apparatus, Holliston, MA, USA). Electroporation settings were adjusted to a single pulse, 400 V, 500 μs. Subsequently, MUC1-DCs were washed 3 times with saline, suspended in 2 ml saline and injected intradermally in the inguinal region (Figure 1b).

### Enhanced green fluorescent protein (EGFP) expression on mDCs by flow cytometry

EGFP mRNA electroporated DCs were checked for EGFP expression 18 h after transfection by an EPICS Flow Cytometer. Gating was performed on cells exhibiting a large forward scatter (FSC) and side scatter (SSC) profile in order to allow exclusion of contaminating autologous lymphocytes. Gated DCs were then evaluated for EGFP expression. Non-transfected DCs were used as a control.

### Analysis of DC subsets

Induced DC subsets were analyzed with mAbs against surface antigens. All mAbs were purchased from Coulter (Hialeah, FL, USA). FITC-conjugated anti-CD80 (B7-1), -CD83 (HB-15), -CD14 (B1), -HLA-ABC and –HLA-DR (I2) were used. PE-conjugated anti-CD86 (B7-2) and -CD40 were also used according to the manufacturer's instructions. Samples were analyzed with an EPICS Flow Cytometer (Coulter Electronics, Inc., Hialeah, FL, USA) at a fluorescence excitation wavelength of 488 nm at 200–500 mW. For each sample, 5,000 DCs were analyzed.

### Analyses of Myeloid-derived suppressor cell (MDSC) and regulatory T cell (Treg) in PBMCs

PBMCs (obtained before the treatment and one month after 3 transfers) were enriched by density gradient centrifugation with Ficoll-Paque (Amersham Pharmacia Biotech, Uppsala, Sweden). Cells were aliquoted for MDSC and Treg analysis. Cells were incubated with energy-coupled dye-phycoerythrin-Texas Red (ECD)- conjugated anti-human CD4 (T4), FITC-conjugated anti-human CD25 (Beckman Coulter), VioBlue-conjugated anti-human CD11b (M1/70.15.11.5) (Miltenyi Biotech, Bergisch Gladbach, Germany), FITC-conjugated anti-human CD33 (HIM3-4) (eBioscience, San Diego, CA, USA) or the corresponding isotype control Abs (Beckman Coulter) for 30 min at 4°C. For Treg intra-nuclear Foxp3 analysis, after treatment with rat serum and permeabilization buffer for 15 min at 4°C, cells were incubated with rat PE-labeled human Foxp3 Ab (PCH101) (eBioscience, San Diego, CA, USA) or the appropriate isotype control (Beckman Coulter) for 30 min at 4°C, then washed, re-suspended in 1% paraformaldehyde (PFA) in Dulbecco’s phosphate-buffered saline (D-PBS) (Nissui pharmaceutical, Tokyo, Japan), and stored at 4°C in the dark until flow cytometric analysis. Two-color flow cytometry was performed with an EPICS flow cytometer (Coulter Electronics, Inc., Hialeah, FL). Treg analysis was performed using at least 30,000 cells that were gated in the region of the lymphocyte population, whereas for MDSC analysis, all cells including the region of mononuclear cells and the polymorphonuclear leukocyte population were analyzed. Lymphocytes were gated in FSC and SSC profiles. Tregs were identified as CD4 + CD25+ Foxp3+ and calculated as a percentage of CD4+ lymphocytes. MDSCs were identified as CD11b + CD33+ [30] and calculated as a percentage of total PBMC.

### Enzyme-linked immunoSpot (ELISPOT) assay

Frozen PBMCs, obtained before the treatment, after first treatment and after third treatment, were thawed prior to use and rested overnight in 10 U/ml benzonase nuclease (Novagen) at 37°C 5% CO_2_ in a humidified incubator, and then used in the next step. PBMCs (responder cells) were cultured with the MUC1-mRNA electroporated PBMCs (stimulator cells) at the responder cells to stimulator cells (R/S ratio) of 1:1, and then were measured for interferon-γ (IFN-γ) responses using ex vivo ELISPOT assay. Nitrocellulose bottomed 96-well Multiscreen plates (Millipore, UK Ltd) were coated with anti-human-IFN-γ mAb (Mabtech, UK) overnight at 4°C. PBMCs were plated in 100 μl final volume and plates were incubated for 18–20 h in a 5% CO_2_ atmosphere at 37°C. Assays were performed in triplicate and the results were averaged. Plates were washed and developed. Number of spots on the plate was counted by Eliphoto Scan (Minerva Tech, Tokyo, Japan). MUC1 specific spots of IFN-γ were counted and calculated as described below.

$$\begin{array}{ll} \mathrm{MUC}1\;\mathrm{specific}\;\mathrm{spots}\;= & \left(\mathrm{Number}\;\mathrm{of}\;\mathrm{spots}\;\mathrm{from}\;A\right)\\ & ‒\left(\mathrm{Number}\;\mathrm{of}\;\mathrm{spots}\;\mathrm{from}\;B\right) \end{array}$$

A; PBMCs co-cultured with PBMCs electroporated with MUC1 mRNA

B; PBMCs co-cultured with PBMCs electroporated without mRNA

### Evaluation of DC migration

Indium oxine (^111^In-Oxine) labeled DC study was performed in one patient with stable disease (SD). DCs were labeled according to the protocols supplied by the manufacturer (Nihon Medi-Physics, Hyogo, Japan). Mature-DCs were resuspended in platelet-poor autologous plasma (CFP1) and incubated for 15 min at room temperature with radioactive ^111^In-Oxine (1 mCi) (Nyconmed Amersham Inc., Buckinghamshire, UK). After two washes to eliminate the unbound isotope, the cells were resuspended in a total volume of 1.5 ml of CFP1. Radiolabelling of the DCs and culture supernatant was evaluated with a gamma camera, after which DCs were intradermally inoculated 10 cm from inguinal lymph nodes. Scintigraphic images of the depot were acquired with a gamma camera 0, 2, 24, and 48 h after injection.

### Statistical analysis

Results are expressed as means ± standard error (SE). All data were analyzed by using GraphPad Prism V5.0 (GraphPad Software, Inc., San Diego, CA). Changes in surface markers were assessed with the paired Student’s *t* test. Survival curves were analyzed by the Kaplan-Meier method and the log-rank test. Categorical variables were compared by using Chi-square and Fisher’s exact test. P-values <0.05 were considered statistically significant.

## Results

### Clinical outcomes

**Figure 2:**
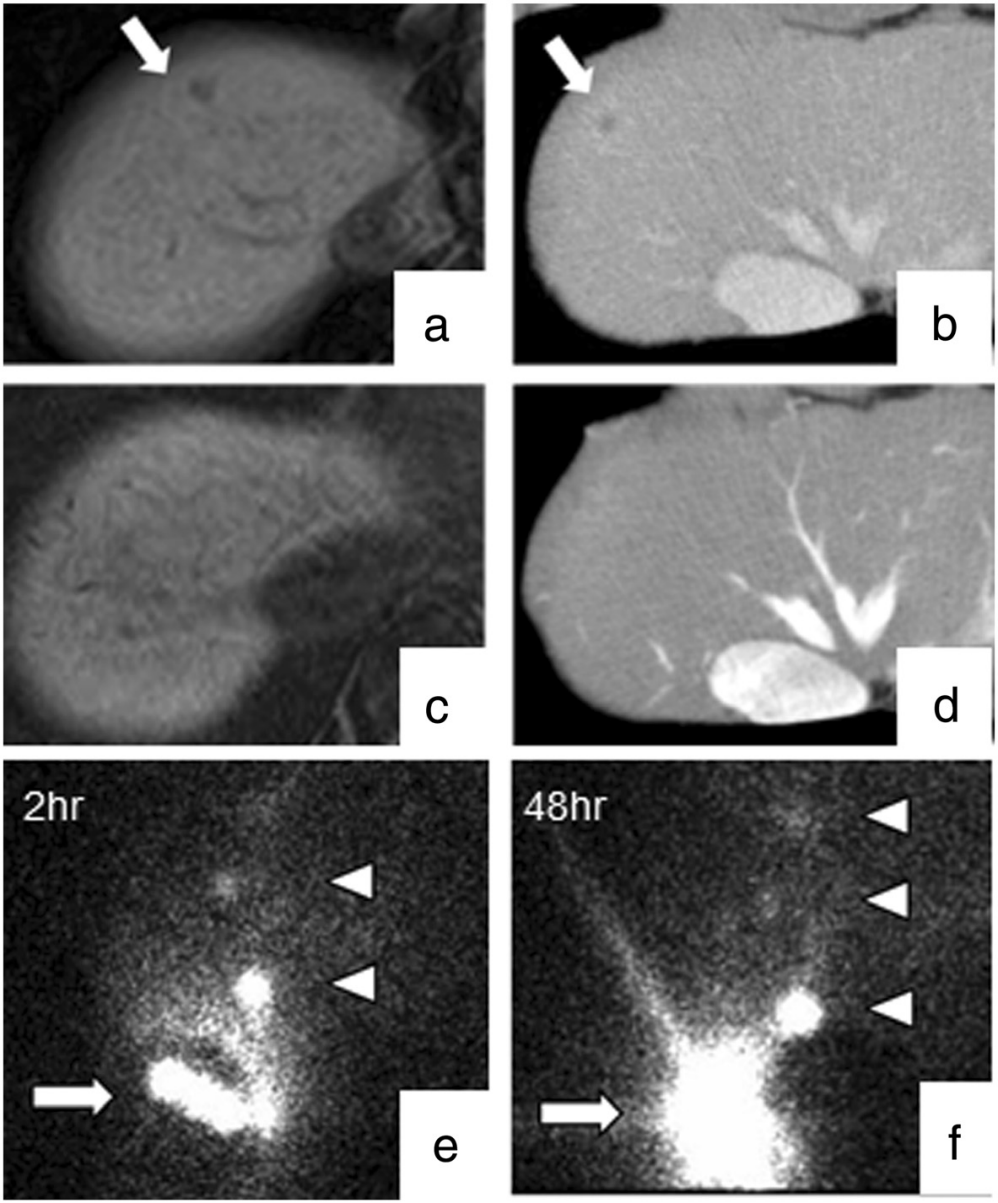
**MRI (Gd-EOB-DTPA MRI, hepatobiliary phase) and CT scans of a patient with CR. Monitoring of in vivo migration of **^**111**^**In-oxine labeled dendritic cells with scintigraphy.** MRI and CT scans revealed a liver metastatic lesion (arrow) **(a, b)**. After 3 cell transfers, the metastatic lesion disappeared completely **(c, d)**. **(a, c)**: MRI. **(b,d)**: CT. Two hours after injection, DCs migrated from the injection site to inguinal lymph nodes (arrowhead) **(e)**. Forty-eight hours after injection, that accumulation extended into distant lymph node, but still remained in the injection site (arrow) and inguinal lymph nodes **(f)**.

Patient characteristics and clinical outcomes are summarized in Table 1. Of 42 patients receiving AIT with MUC1-DCs and MUC1-CTLs plus GEM, 1 patient with recurrence had complete response (CR) (2.4%), 3 patients with stage III (n = 1) and stage IV (n = 2) had partial response (PR) (7.1%), 22 patients with stage III (n = 11), stage IV (n = 7) and recurrence (n = 4) had SD (52.4%), and 16 patients with stage III (n = 2), stage IV (n = 10) and recurrence (n = 4) had PD (38.1%). The disease control rate was 61.9%.Images from gadolinium ethoxybenzyl diethylenetriamine pentaacetic acid-enhanced (Gd-EOB-DTPA) MRI and CT scans of a patient with CR are shown in Figure 2. He had liver metastasis after curative surgery (Figure 2a and b). After 3 transfers, liver metastasis disappeared completely (Figure 2c and d). In contrast, the other 6 patients who had liver metastasis before this therapy had PD and the median survival time (MST) was 6.3 months (data not shown).

**Table 1:** Patient characteristics and clinical outcomes

Number of patients	42
Age (years)	
Mean	63.1
Range	37-81
Sex	
Males	21
Females	21
Stage	
III	14
IV	19
Recurrence	9
Liver metastasis (pretreatment)	
Absence	35
Presence	7
HLA	
HLA-A24	24
Others	17
Unknown	1
Administration (times)	
< 3	22
≧ 3	20
Number of CTLs (×10^8^/time)	
Mean	6.3
Range	1.0-12.4
Number of DCs (×10^7^/time)	
Mean	1.8
Range	0.04-3.9
Prior therapy	
None	25
Tumor resection	9
Chemotherapy	16
Radiotherapy	0
Response	
CR	1
PR	3
SD	22
PD	16

HLA; human leukocyte antigen, CTL; cytotoxic T lymphocyte,DC; dendritic cell, CR; complete response, PR; partial response,SD; stable disease, PD; progressive disease.

**Figure 3:**
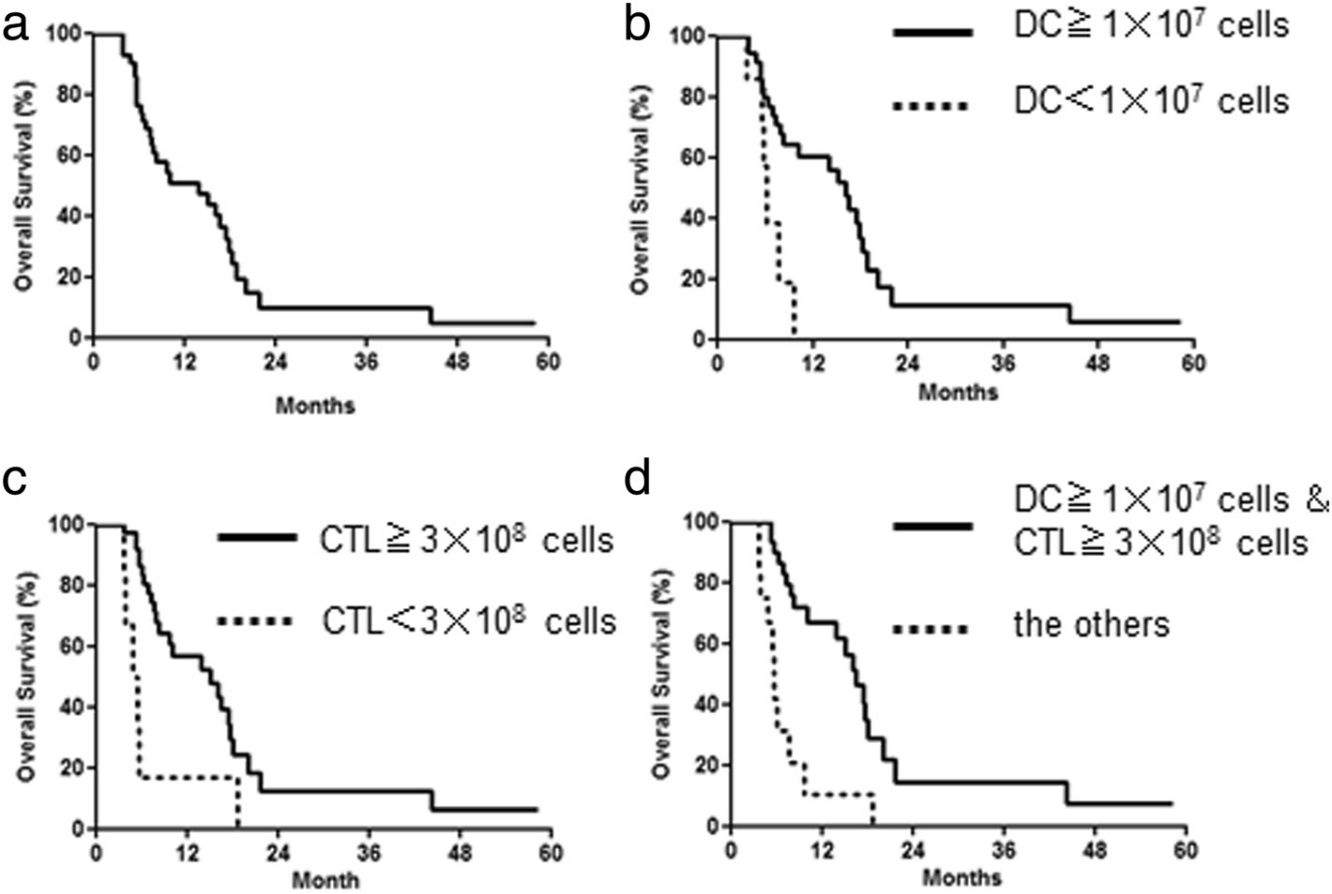
**Overall survival rates in patients with advanced pancreatic cancer treated by AIT with MUC1-DCs and MUC1-CTLs plus GEM. a**; MST was 13.9 months, and the 1-year survival rate was 51.1% in all 42 patients. **b**; 35 patients who received more than 1 × 10^7^MUC1-DCs per injection (MST, 16.1 months) vs. 7 patients who received less than 1 × 10^7^cells (MST, 6.2 months), p = 0.0036. **c**; 36 patients who received more than 3 × 10^8^ MUC1-CTLs per injection (MST, 15.1 months) vs. 6 patients who received less than 3 × 10^8^ (MST, 5.2 months), p = 0.0060. **d**; 30 patients who received both more than 1 × 10^7^MUC1-DCs per injection and 3 × 10^8^ MUC1-CTLs per injection (MST, 16.5 months) vs. the other 12 patients (MST, 5.7 months), p = 0.00020.

The 1-year survival rate was 51.1%, and the MST was 13.9 months in all patients (Figure 3a). Liver metastasis during therapy appeared in only 5 of 35 patients without liver metastasis before treatment. The 1-year survival rate and MST in 35 patients who received more than 1 × 10^7^ MUC1-DCs per injection were significantly better than for 7 patients who received less than 1 × 10^7^cells (60.3% vs. 0%, 16.1 months vs. 6.2 months, p = 0.0036) (Figure 3b). Administration times and total number of MUC1-CTLs and MUC1-DCs were 6.2 ± 0.8 times, 4.5 ± 0.7 × 10^9^ cells and 12.9 ± 1.8 × 10^7^ cells in the high dose group, and 3.1 ± 0.2 times, 1.5 ± 0.2 × 10^9^ cells and 1.9 ± 0.5 × 10^7^ cells in the low dose group. The 1-year survival rate and MST in 36 patients who received more than 3 × 10^8^ MUC1-CTLs per injection were significantly better than for 6 patients who received less than 3 × 10^8^ cells (56.6% vs. 16.7%, 15.1 months vs. 5.2 months, p = 0.0060) (Figure 3c). Administration times and total number of MUC1-CTLs and MUC1-DCs were 6.3 ± 0.8 times, 4.6 ± 0.7 × 10^9^ cells and 12.4 ± 1.8 × 10^7^ cells in the high dose group, and 2.3 ± 0.3 times, 0.5 ± 0.1 × 10^9^ cells and 2.4 ± 0.5 × 10^7^ cells in the low dose group. The 1-year survival rate and MST in 30 patients who received both more than 1 × 10^7^MUC1-DCs per injection and 3 × 10^8^ MUC1-CTLs per injection were significantly better than for the other 12 patients (66.7% vs. 10.4%, 16.5 months vs. 5.7 months, p = 0.0002) (Figure 3d). Administration times and total number of MUC1-CTLs and MUC1-DCs were 6.8 ± 0.9 times, 5.0 ± 0.8 × 10^9^ cells and 14.4 ± 2.0 × 10^7^ cells in the high dose group, and 2.9 ± 0.3 times, 1.3 ± 0.3 × 10^9^ cells and 2.5 ± 0.5 × 10^7^ cells in the low dose group. There was no significant bias of patient characteristics between the high dose group and low dose group (Table 2).

**Table 2:** Patient characteristics between the high dose group and low dose group

	**DC**			**CTL**		
	**High dose group**	**Low dose group**	**p-value**	**High dose group**	**Low dose group**	**p-value**
	**(n = 35)**	**(n = 7)**		**(n = 36)**	**(n = 6)**	
Stage			p = 0.3053			p = 0.0976
III	12	2		14	0	
IV	17	2		14	5	
Recurrence	6	3		8	1	
Liver metastasis (pretreatment)			p = 0.5788			p = 1.000
Absence	30	5		30	5	
Presence	5	2		6	1	

DC; dendritic cell, CTL; cytotoxic T lymphocyte.

A comparative analysis revealed that there were no significant differences between the clinically good (CR/PR/SD) and poor responders (PD) for age, sex, disease stage and neutrophil/lymphocyte ratio, except for pretreatment liver metastasis (p = 0.0081) and the number of DCs (p = 0.0015). The number of CTLs per injection was trend to higher in the clinically good responders (p = 0.0537) (Table 3).

**Table 3:** Comparison between the clinically good and poor responders

**Characteristics**	**Clinically good (CR/PR/SD)**	**Clinically poor (PD)**	**p-value**
Number of patients	26	16	
Age (years)	61.6 (37-81)	65.6 (43-81)	p = 0.2434
Sex			p = 0.7513
Males	12	9	
Females	14	7	
Stage			p = 0.0735
III	12	2	
IV	9	10	
Recurrence	5	4	
Liver metastasis (pretreatment)			p = 0.0081
Absence	25	10	
Presence	1	6	
NLR	2.3 (0.7-6.4)	3.1 (0.4-6.1)	p = 0.1364
Number of CTLs (x10^8^/time)	7.0 (2.8-12.4)	5.1 (1.0-9.0)	p = 0.0537
Number of DCs (x10^7^/time)	2.1 (0.9-3.9)	1.2 (0.04-2.9)	p = 0.0015

CR; complete response, PR; partial response, SD; stable disease, PD; progressive disease NLR; neutrophil/lymphocyte ratio, CTL; cytotoxic T lymphocyte, DC; dendritic cell.

### Safety and toxicity

The major grade 3 and 4 adverse events are summarized in Table 4. The most common grade 3or 4 hematologic adverse event was neutrophils (31%). Grade 3 or 4 anorexia (14.3%) was the most nonhematologic adverse event. No AIT-related adverse events such as rash, fever, chill, and injection site reaction were observed. There was no clinical or radiological evidence of autoimmune reaction in any of the patients.

**Table 4:** Grade 3 and 4 Adverse Events in 42 patients

**Adverse event**	**Number of patients**	**%**
Leukocytes	11	26.2
Neutrophils	13	31
Platelets	1	2.4
Hemoglobin	5	11.9
AST	1	2.4
ALT	1	2.4
Fatigue	5	11.9
Anorexia	6	14.3
Diarrhea	3	7.1
Mucositis/stomatitis	1	2.4
Nausea	3	7.1
Vomiting	1	2.4

AST; aspartate aminotransferase, ALT; alanine aminotransferase.

### Cytotoxic activity of induced MUC1-CTLs and antibody inhibition of cytotoxicity

Induced MUC1-CTLs (HLA-A24/26) showed strong cytotoxicity against pancreatic cancer cell lines (YPK-1; HLA-A24, and YPK-3; HLA-A02) which expressed MUC1 antigen on the cell surface in an HLA- unrestricted manner. However, cytotoxicity against the esophageal cancer cell lines (YES-1 and -2), which did not express MUC1, was low (Additional file 1: Figure S1a).

Anti-CD3 mAb or anti-CD8 mAb inhibited CTL cytotoxicity against YPK-1 cells. Anti-MUC1 mAb also inhibited cytotoxicity in these cells. Anti-class I mAb showed no inhibition of CTL cytotoxicity (E:T = 20:1; anti-CD3, 66.5%; -CD4, 26.9%; -CD8, 76.9%; anti-class I, 11.8% and anti-MUC1, 64.1%) (Additional file 1: Figure S1b) [15]. These results showed that cytotoxicity of induced MUC1-CTLs was MUC1 specific and HLA-unrestricted.

### EGFP expression in EGFP mRNA electroporated DCs

**Figure 4:**
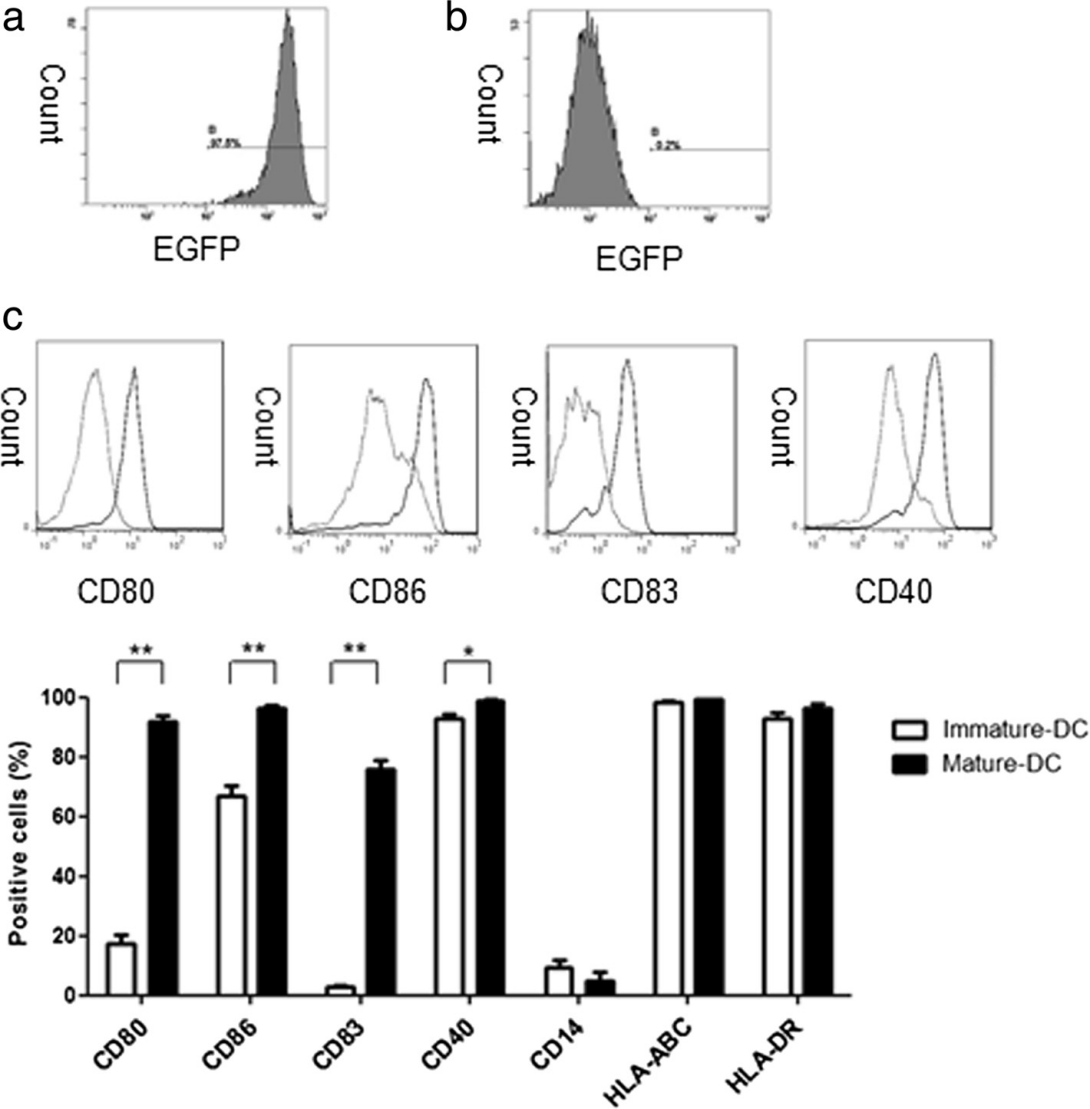
**Flow cytometric analysis of DCs.** EGFP expression was analyzed by flow cytometry 18 h post-transfection. **a**; The expression level of EGFP was more than 95%. **b**; As a control non-transfected DC were used. **c**; Expression of DC surface markers was evaluated by flow cytometry. Twenty nine patients were evaluable. Comparative data were shown in histograms for immature (dotted line) and mature (solid line) DCs. The expression of each antigen in imDCs was found in 17.5 ± 2.9% (CD80), 67.1 ± 3.1% (CD86), 2.8 ± 0.7% (CD83), 92.6 ± 1.7% (CD40), 9.5 ± 2.3% (CD14), 98.6 ± 0.3% (HLA-ABC) and 92.8 ± 1.8% (HLA-DR). The expression of each antigen in mDCs was found in 92.1 ± 1.5% (CD80), 96.5 ± 0.7% (CD86), 75.6 ± 3.3% (CD83), 98.9 ± 0.5% (CD40), 4.6 ± 3.4% (CD14), 99.4 ± 0.2% (HLA-ABC) and 96.6 ± 1.1% (HLA-DR). *p = 0.0004, **p < 0.0001.

To confirm the translation efficiency from introduced mRNA to protein, the mDCs were electroporated with EGFP mRNA. The expression coefficient of EGFP was more than 95% (Figure 4a). This result indicates that DCs can translate the introduced mRNAs to encoded protein.

### Profiles of surface markers of induced DCs

A comparison of surface marker expression between imDCs and mDCs was shown (Figure 4c). Twenty nine patients were evaluable. The expression of each antigen in imDCs was found in 17.5 ± 2.9% (CD80), 67.1 ± 3.1% (CD86), 2.8 ± 0.7% (CD83), 92.6 ± 1.7% (CD40), 9.5 ± 2.3% (CD14), 98.6 ± 0.3% (HLA-ABC) and 92.8 ± 1.8% (HLA-DR). The expression of each antigen in mDCs was found in 92.1 ± 1.5% (CD80), 96.5 ± 0.7% (CD86), 75.6 ± 3.3% (CD83), 98.9 ± 0.5% (CD40), 4.6 ± 3.4% (CD14), 99.4 ± 0.2% (HLA-ABC) and 96.6 ± 1.1% (HLA-DR). The percentage of CD80+, CD83+, and CD86+ DCs was extremely increased in mDCs as compared with imDCs (P < 0.0001). A high expression level of CD40 was also observed in mDCs (P = 0.0004). HLA-class I and HLA-class II expression levels were identical between imDCs and mDCs.

### Change of CD11b + CD33+ cells and CD4+ CD25+ Foxp3+ cells in PBMCs

**Figure 5:**
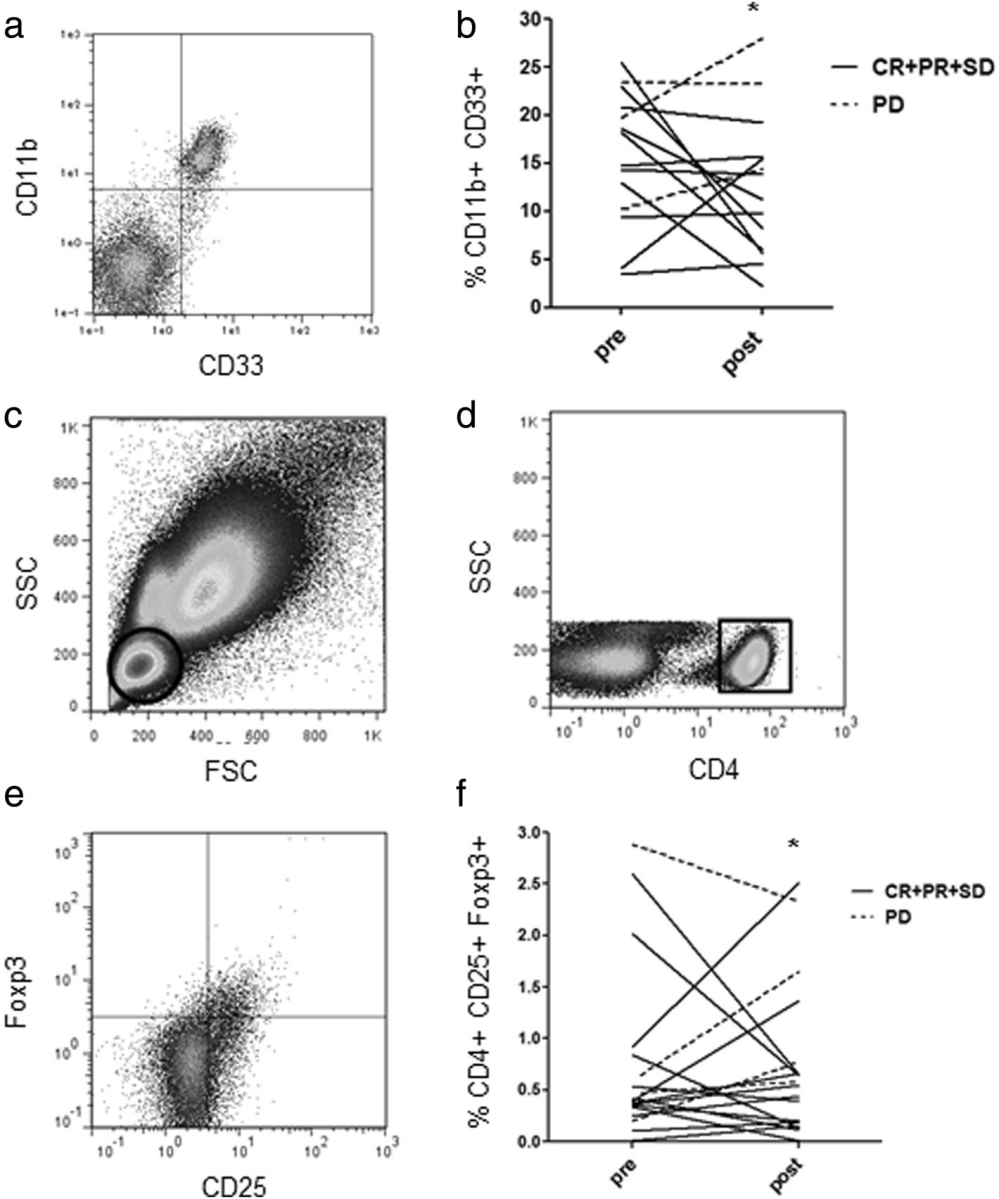
**Frequency of CD11b + CD33+ cells and CD4 + CD25 + Foxp3+ cells in the PBMCs. a**; Analysis for CD11b as well as CD33 expression by flow cytometry. **b**; The percentage of CD11b + CD33+ cells of patients with CR, PR and SD (n = 11) (solid line) and PD (n = 3) (dashed line) was measured. *p = 0.043. **c**; Lymphocytes were identified based on their characteristic properties shown in the FSC and SSC. **d**; A representative gating was set for CD4+ cells from lymphocytes. **e**; Analysis for CD25 as well as Foxp3 expression in the CD4+ lymphocytes gate. **f**; The percentage of CD4 + CD25 + Foxp3+ cells of patients with CR, PR and SD (n = 14) (solid line) and PD (n = 4) (dashed line) was measured. * p = 0.0495.

We assessed negative immune factors focusing on CD11b + CD33+ cells (n = 14) and CD4+ CD25+ Foxp3+ cells (n = 18) in PBMCs before and one month after 3 transfer. Before treatment there was no difference in the percentage of CD11b + CD33+ cells between patients with CR, PR, and SD (15.1 ± 2.2, n = 11) and patients with PD (17.8 ± 3.9, n = 3). After treatment, the percentage of CD11b + CD33+ cells in patients with CR, PR and SD (10.2 ± 1.6) was significantly lower than patients with PD (21.9 ± 4.0) (p = 0.0430) (Figure 5b). Before treatment there was no difference in the percentage of CD4+ CD25+ Foxp3+ cells between patients with CR, PR and SD (0.68 ± 0.20, n = 14) and patients with PD (1.03 ± 0.62, n = 4). After treatment, the percentage of CD4+ CD25+ Foxp3+ cells in patients with CR, PR and SD (0.57 ± 0.18) was significantly lower than patients with PD (1.33 ± 0.41) (p = 0.0495) (Figure 5f).

### ELISPOT analysis

**Figure 6:**
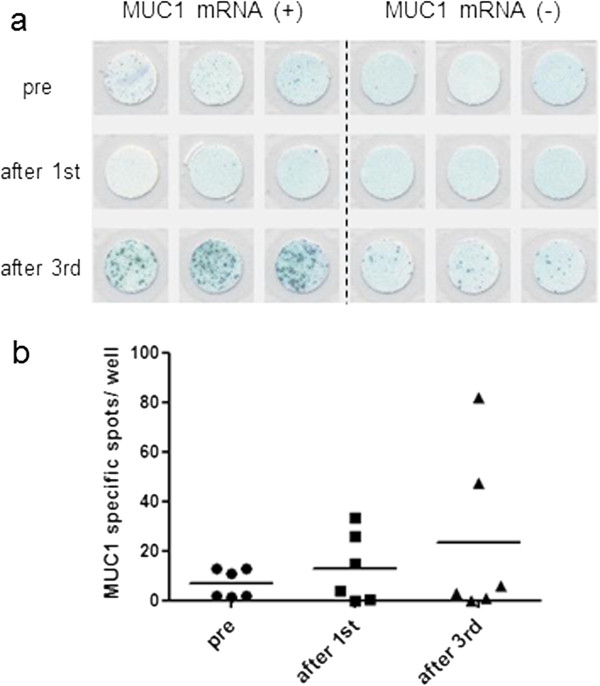
**Representative immunologic monitoring assays detecting antigen-specific responses induced IFN-γ producing cells. a**; IFN-γ producing cells were increased in PBMCs co-cultured with PBMCs electroporated with MUC1 mRNA compared with PBMCs co-cultured with PBMCs electroporated without MUC1 mRNA. **b**; Six patients were evaluable. More MUC1 specific spots were observed in post-treatment compared with pretreatment PBMC samples.

IFN-γ ELISPOT assay was performed to evaluate the specific responses to MUC1 of immune effector cells from 6 patients. The number of MUC1 specific spots was 6.8 ± 2.3, 13.1 ± 5.8 and 23.2 ± 13.9, prior to treatment, after first treatment and after third treatment, respectively. More IFN-γ spots were observed in post-treatment compared with pretreatment PBMC samples. Although IFN-γ activity was increased in this assay, it was still unclear which cells had high activity such as CTL or NK cells (Figure 6).

### Monitoring of in vivo migration of ^111^Indium (In)-oxine labeled dendritic cells with scintigraphy

Scintigraphic images obtained from one patient 2 h and 48 h after DC administration demonstrated that ^111^In-oxine labeled DCs accumulated at the injection site and regional lymph nodes 2 h after injection. Forty-eight hours after injection, the accumulation extended into distant lymph nodes, but still remained in the injection site and regional lymph node (Figure 2e and f).

## Discussion

In the present study, adoptive immunotherapy (AIT) with MUC1-DCs and MUC1-CTLs plus GEM resulted in a 1-year survival rate of greater than 50% in patients with unresectable or recurrent pancreatic invasive ductal carcinoma (Figure 3a). One patient, who had liver metastasis after curative surgery, had CR (Figure 2a to d). Three patients had PR, and one of these patients had curative surgery 12 months after this therapy. Although it have been reported that 73% of patients who died from pancreatic cancer were found to present with liver metastases at autopsy [31], in this therapy liver metastasis appeared in only 5 patients (14%) among 35 patients without liver metastasis before treatment. We observed no severe adverse events related to our AIT (Table 4).

MST following GEM monotherapy, which is the standard chemotherapy for unresectable pancreatic cancer, was 5.7 months, and the 1-year survival rate was 18% [24]. Although some trials of combination therapies including GEM and other cytotoxic agents resulted in improved response rates over GEM alone, they failed to show survival benefits [32-34]. The combination of erlotinib plus GEM showed a significant improvement in overall survival; however, the increase in MST was marginal (6.24 vs. 5.91 months) [35]. In another study, MST was 11.1 months for the FOLFIRINOX group, compared with 6.8 months in the GEM group, showing a significant difference. However, markedly more adverse events were noted in the FOLFIRINOX group [36]. Since these outcomes in advanced pancreatic cancer are still poor, more effective treatment strategies are required.

We have previously used DCs pulsed with MUC1 peptide [16]. In this study, mDCs were transfected with MUC1-mRNA by electroporation, because antigen epitopes are naturally processed, and a variety of different epitopes are long term presented by both HLA class I and class II molecules. It has been reported the advantage of endogenous expression by DC is that T cell epitopes do not need to be specified, HLA type is not a limiting factor and multiple epitopes (both CTL and helper T cell epitopes) can be presented [37]. In our previous therapy (MUC1 peptide-pulsed DCs and MUC1-CTLs), the MST was 9.8 months [16]; in the present study, the MST was 13.9 months. The improved survival benefit of the present study may be related to patient characteristics such as distant metastasis, the GEM combination, or MUC1 mRNA transfection. Distant metastasis such as liver metastasis, lung metastasis or peritoneal dissemination was present in 15 of 20 (75%) previous patients and 28 of 42 (66.7%) present patients, which is no significant difference.

GEM has the potential to augment the antitumor effects of cancer immunotherapy by suppressing Treg induction [25,38], and also reduces MDSC [39], but does not reduce CD4+ T cells, CD8+ T cells, NK cells, macrophages, or B cells [40]. We therefore performed a combination therapy with AIT and GEM. In the present study, we observed a significant decrease in the percentages of MDSC (Figure 5b, p = 0.043) and Treg (Figure 5f, p = 0.0495) in patients with CR, PR and SD when compared with those in patients with PD. Hence, we speculated that clinical benefit may be related to the reductions of MDSC and Treg.

A survival and clinical benefit was shown in the patients who received high dose MUC1-DCs and MUC1-CTLs per injection (Figure 2b to 3d, Table 3). Administration of a large number of DCs and CTLs may be necessary to achieve a clinical effect. Induction of CTLs in patients with advanced pancreatic cancer may be suppressed by activated granulocytes and MDSC [41]. We have reported that pancreatic cancer patients had low CTL precursors reactive to EBV peptide as compared with healthy volunteers [4]. This result suggests that the cellular immunity of these patients might be depressed, and therefore other supportive immunotherapies may be needed for these patients to increase their general level of immunity prior to specific immunotherapy.

The migration of DC is an important question that needs to be addressed in clinical therapy. A key step involved with T cell sensitization after administration is DC migration into regional draining lymph nodes and antigen presentation to lymphocytes. In the present study, the induction of mDCs was performed successfully, which was demonstrated by the high expression of CD83 (75.6 ± 3.3%, Figure 4c) that expressed at activated and mDCs [42]. It has been reported that a better migration activity is obtained using intradermal route than subcutaneous routes and that mDCs show higher migration than imDCs [43,44]. We confirmed the migration of administered mDCs by scintigraphic images collected 2 and 48 h after injection in one patient. Two hours after injection, mDCs migrated from the injection site to inguinal lymph nodes. Forty-eight hours after injection, that accumulation extended into distant lymph node, but still remained in the injection site as well as inguinal lymph nodes (Figure 2e and f).

In conclusion, our cancer immunotherapy in combination with GEM was safe and appears to be effective for the patients with unresectable pancreatic cancer. Although we need to verify this preliminary result by a much larger prospective randomized study, we believe that these findings surely lead to the novel therapeutic strategy for advanced pancreatic cancer.

## Conclusions

We retrospectively analyzed the outcome of 42 patients with unresectable or recurrent pancreatic cancer treated with MUC1-DCs (MUC1-mRNA transfected DCs) and MUC1-CTLs (lymphocytes stimulated by co-culture with a MUC1 expressing human pancreatic cancer cell line and IL-2). Our adoptive immunotherapy was safe and effective in a subgroup with sufficient induction of DCs and CTLs. Further randomized control studies of large numbers of patients are needed to confirm the efficacy of this combination therapy for unresectable pancreatic cancer.

## Abbreviations

AIT: Adoptive immunotherapy; CR: Complete response; CT: Computed tomography; CTCAE: Common Terminology Criteria for Adverse Events; CTLs: Cytotoxic T lymphocytes; DCs: Dendritic cells; ECOG: Eastern Cooperative Oncology Group; EGFP: Enhanced green fluorescent protein; ELISPOT: Enzyme-Linked ImmunoSpot; FSC: Forward scatter; Gd-EOB-DTPA: Gadolinium ethoxybenzyl diethylenetriamine pentaacetic acid; GEM: Gemcitabine; GM-CSF: Granulocyte macrophage colony-stimulating factor; HLA: Human leukocyte antigen; IFN-γ: Interferon-γ; IL-2: Interleukin 2; imDC: Immature DCs; ^111^In-Oxine: Indium oxine; LAK: Lymphokine-activated killer; mAbs: Monoclonal antibodies; mDCs: Mature DCs; MDSC: Myeloid-derived suppressor cell; MRI: Magnetic resonance imaging; MST: Median survival time; MUC1: Mucin 1; PBMCs: Peripheral blood mononuclear cells; PD: Progressive disease; PR: Partial response; PS: Performance status; RECIST: Response evaluation criteria in solid tumors; SD: Stable disease; SE: Standard error; SSC: Side scatter; TNF-α: Tumor necrosis factor-α; Treg: Regulatory T cell; U: Units.

## Competing interests

The authors declare that they have no competing interests.

## Authors’ contributions

YS performed and evaluated the study, and wrote the manuscript. SH designed this clinical study and participated in review and revision of the manuscript. YM, HM, MI, NS, KY, TU, SY, KS, YS, TY and YH assisted to perform the study and data analysis. MO participated as principle investigator of the study, and drafted the manuscript. All authors read and approved the final manuscript.

## Supplementary Material

Additional file 1: Figure S1 Cytotoxicity of induced MUC1-CTLs and antibody inhibition of cytotoxicity. **(a)** CTLs were stimulated by the MUC1-expressing human pancreatic cancer cell line, YPK-1 (HLA-A 2402, MUC1-positive). Target cell lines were YPK-1, YPK-3 (pancreatic cancer, HLA-A 0201, MUC1-positive), YES-2 (esophageal cancer, HLA-A 2404, MUC1-negative) and YES-1 (esophageal cancer, HLA-A 0201, MUC1-negative). Induced CTLs were cytotoxic against MUC1-expressing pancreatic cancer cell lines regardless of the HLA-A phenotype. Low cytotoxicity was observed in MUC1-negative esophageal cancer cell lines. Cytotoxicity was MHC unrestricted and clearly decreased with the decreasing effector cell number. **(b)** Anti-CD3 or -CD8 mAb strongly inhibited cytotoxicity against YPK-1cells, whereas anti-class I mAb showed no inhibition. YPK-1 cells treated with anti-MUC1 mAb also showed a low cytotoxicity. Click here for file
