# Supplementary figures and images for: Adoptive immunotherapy with MUC1-mRNA transfected dendritic cells and cytotoxic lymphocytes plus gemcitabine for unresectable pancreatic cancer
Source: J Transl Med. 2014 Jun 19;12:175. doi: 10.1186/1479-5876-12-175 (PMC4074851; doi:10.1186/1479-5876-12-175)

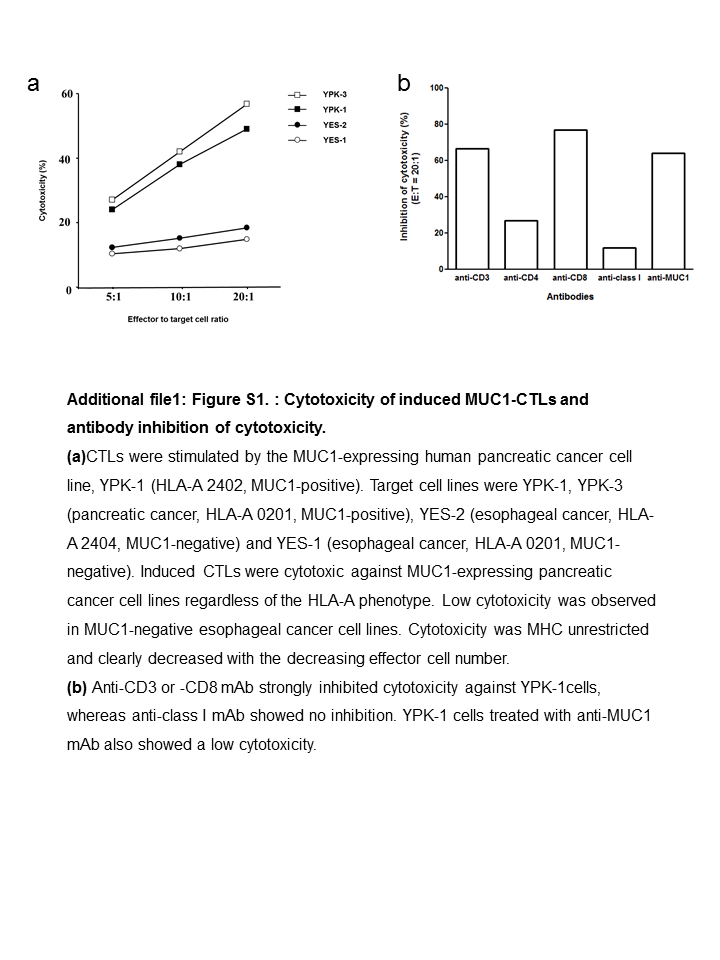

Supplement: Additional file 1: Figure S1 — Cytotoxicity of induced MUC1-CTLs and antibody inhibition of cytotoxicity. (a) CTLs were stimulated by the MUC1-expressing human pancreatic cancer cell line, YPK-1 (HLA-A 2402, MUC1-positive). Target cell lines were YPK-1, YPK-3 (pancreatic cancer, HLA-A 0201, MUC1-positive), YES-2 (esophageal cancer, HLA-A 2404, MUC1-negative) and YES-1 (esophageal cancer, HLA-A 0201, MUC1-negative). Induced CTLs were cytotoxic against MUC1-expressing pancreatic cancer cell lines regardless of the HLA-A phenotype. Low cytotoxicity was observed in MUC1-negative esophageal cancer cell lines. Cytotoxicity was MHC unrestricted and clearly decreased with the decreasing effector cell number. (b) Anti-CD3 or -CD8 mAb strongly inhibited cytotoxicity against YPK-1cells, whereas anti-class I mAb showed no inhibition. YPK-1 cells treated with anti-MUC1 mAb also showed a low cytotoxicity. [file 1479-5876-12-175-S1.tiff]
